# Will Roe Deer Persist in a Warmer World?

**DOI:** 10.1371/journal.pbio.1001829

**Published:** 2014-04-01

**Authors:** Jonathan Chase

**Affiliations:** Freelance Science Writer, Saint Louis, Missouri, United States of America

[Fig pbio-1001829-g001]The Earth's climate is rapidly changing at the hands of human activities. There is little doubt about this. There is doubt, however, about how Earth's inhabitants will respond to this change. By combining models of expected climate change with models that match climates to the distributions of species, ecologists can project scenarios of future biodiversity. These projections are usually rather bleak. More recently, however, many ecologists have questioned the gross oversimplifications that go into some of these projections, such as the supposition that species are largely immutable, unwavering in their behavior, physiology, and other traits despite rapidly changing environs.

**Figure pbio-1001829-g001:**
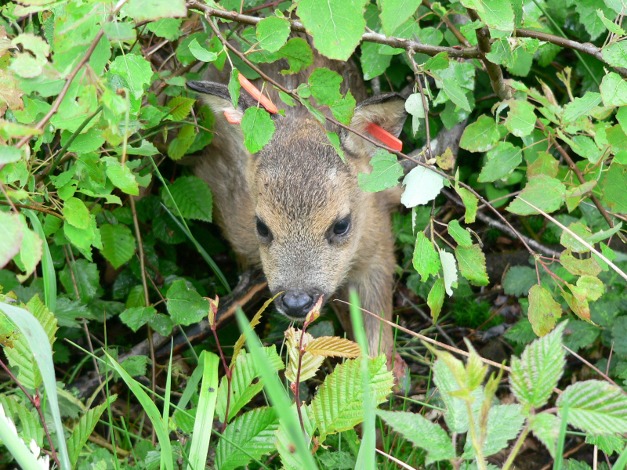
Increasing mismatch between birth date and earlier spring vegetation means the probability of survival of this fawn born in May 2005 is lower than that of a fawn born 20 years previous. *Image credit: Gilles Bourgoin*

To more directly evaluate species' responses to climate change, some ecologists have taken advantage of long-term, often serendipitous, observations. For example, Henry David Thoreau's meticulous observations at Walden Pond have allowed scientists to show that the most common spring wildflowers are blooming 10 days earlier in the spring, on average, and the leaves on shrubs and trees emerge 18 days earlier, on average, than they did 150 years ago. Results from these, as well as other less literary examples, paint a different picture of ecological responses to global change. Rather than being immutable, the traits of many species change in response to changing climates. These changes include short-term “plastic” shifts, such as the earlier spring emergence of plants and animals, as well as longer-term shifts in population-level traits that result from evolutionary adaptations. Regardless of whether it is due to plasticity or evolution, the ability of species to alter their traits in response to climate change creates doubt about whether the “gloom-and-doom” projections of biodiversity's response to climate change will be realized.

Just because a species can modify its traits in response to climate change does not, however, tell us whether the population trajectory of that species will be influenced. Does the organism's survival and/or reproduction change when climates change? This information is much harder to come by in long-term data. To date, only a handful of long-term studies are available to address the demographic consequences of climate change, most showing that populations can adapt through plasticity or evolutionary adaptation, thereby minimizing the potential negative influence of climate change on population growth. However, because the data used for these studies are rare and often serendipitous, it is unclear whether a lack of evidence for declining populations in the face of climate change is because populations are not generally impacted or simply because there are too few studies available to draw conclusions.

In this issue of *PLOS Biology*, Plard et al. provide evidence to suggest that, at least for the roe deer (*Capreolus capreolus*) of France's Champagne region, climate change is not simply a minor inconvenience to overcome but rather a fundamental impediment to population growth that could portend eventual catastrophe. Plard et al. made use of two long-term datasets: the timing of spring birth and subsequent survivorship of roe deer fawns, and the timing of the flush of nutritious spring vegetation on which roe deer females depend to meet the energetic demands for lactation to feed their fawns. The researchers examined whether and how much the timing of vegetation flush changed as the climate changed, whether any mismatch between the timing of fawn birth relative to the timing of the vegetation flush influenced fawn survivorship, and whether there were any consequences for phenotypic traits of the deer (in either trait plasticity or trait evolution) and/or the fitness and population dynamics of the deer.

The first dataset was collected at Trois Fontaines, an enclosed 1,360-hectare forest where the roe deer population has been intensively monitored for decades. Each spring, biologists systematically capture and tag newborn fawns on the site (from late April to mid-June), noting the date each one was born and the identity of its mother. Each winter (January to March), the deer are captured and identified, providing estimates of individual survivorship.

For the second dataset, Plard et al. did not have access to data on the timing of the spring flush of the vegetation (i.e., young shoots and leaves) that provides critical nutrition for lactating female deer, but instead found the next best thing: data on the timing of grapevine flowering. Centuries of experience have taught French winemakers that the timing of grape flowering and fruiting can play a crucial role in the quality and quantity of fruits produced. As a result, Plard et al. were able to access detailed data on the timing of grapevine flowering each year. It turns out that this is about 1 month after the forest vegetation flush occurs. Thus, by analyzing long-term data on grapevine flowering times, Plard et al. were able to estimate the timing of the forest vegetation flush in the study period. In addition to data on deer and plants, Plard et al. also analyzed spring temperature changes. Over the 27-year period examined in detail, they found that, on average, there was a nearly 1.5°C increase in spring (April to June) temperatures at the site. This directly translated into a more than 2-weeks-earlier shift in the timing of grapevine flowering and spring vegetation flush.

Surprisingly, the average dates in which fawns were born did not change over the course of the study despite the rapid advance of springtime vegetation flush. The mismatch between vegetation flush and the dates on which fawns were born increased through time, and as a result fawn survival decreased, most likely because females could not as effectively utilize the nutrition from the spring flush to provision their fawns with milk.

Even though the deer were not able to alter the timing with which they gave birth in response to earlier vegetation flush (i.e., they were not phenotypically plastic), there was considerable variation in the timing of when individual females gave birth (larger and longer-lived females tended to give birth earlier). There was also strong directional natural selection; individuals born more coincident with the spring vegetation flush were much more likely to survive than those born well after the flush. However, because there was no statistically significant heritability in the timing of births—fawns born earlier in the spring were no more likely to give early spring birth to their own offspring—there was no detectable evolutionary trend in this trait throughout the study period.

As a result of the decreased rates of offspring survival—as the mismatch between birth dates and the spring vegetation flush increased—and the lack of an evolutionary response, Plard et al. were able to calculate declines in both individual fitness and population growth rate of the roe deer population through time. Although not yet declining towards extinction, the lowered growth rate of these deer seems to be attributable to recent climate change and is likely to get worse as the spring vegetation flush continues to advance with global warming.

We are just beginning to understand how human activities are changing the climate, and the consequences of those changes for biodiversity. Early projections that species will uniformly be negatively altered by climate change have given way to the recognition that many complexities, including phenotypic plasticity and short-term evolutionary responses, can buffer populations of many species. However, the study by Plard et al. provides a somber warning that not all species can and will adapt to changing climates, including species such as roe deer that are (or once were) common and play an important functional role in the ecosystem.

**Plard F, Gaillard J-M, Coulson T, Hewison M, Delorme D, et al. (2014) Mismatch Between Birth Date and Vegetation Phenology Slows the Demography of Roe Deer.** doi:10.1371/journal.pbio.1001828

